# First detection of Tetraparvovirus ungulate 1 in diseased cattle (Chinese Simmental) from Hunan province, China

**DOI:** 10.1186/s12985-024-02402-1

**Published:** 2024-06-06

**Authors:** Yin Shi, Hui Tang, Zhi-Jian Zhou, Jing-Ying Liao, Xing-Yi Ge, Chao-Ting Xiao

**Affiliations:** Institute of Pathogen Biology and Immunology, College of Biology, Hunan Provincial Key Laboratory of Medical Virology, Hunan University, Changsha, 410082 China

**Keywords:** Bovine hokovirus, Genotype, Parvovirus, Tetraparvovirus ungulate 1

## Abstract

Tetraparvovirus is an emerging parvovirus infecting a variety of mammals and humans, and associated with human diseases including severe acute respiratory infection and acute encephalitis syndrome. In the present study, a Tetraparvovirus ungulate 1 (formerly known as bovine hokovirus) strain HNU-CBY-2023 was identified and characterized from diseased Chinese Simmental from Hunan province, China. The nearly complete genome of HNU-CBY-2023 is 5346 nt in size and showed genomic identities of 85-95.5% to the known Tetraparvovirus ungulate 1 strains from GenBank, indicating a rather genetic variation. Phylogenetic and genetic divergence analyses indicated that Tetraparvovirus ungulate 1 could be divided into two genotypes (I and II), and HNU-CBY-2023 was clustered into genotype II. This study, for the first time, identified Tetraparvovirus ungulate 1 from domestic cattle from mainland China, which will be helpful to understand the prevalence and genetic diversity of Tetraparvovirus ungulate 1.

**Supplementary Information:**

The online version contains supplementary material available at 10.1186/s12985-024-02402-1.

Parvoviruses in family *Parvoviridae* are small, non-enveloped, single-stranded DNA viruses, with a genome size of approximately 4,000 to 6,000 nucleotides (nt) that contain terminal palindromic sequences, and the three major open reading frames (ORFs) encode large nonstructural protein (NS1) and virus particle protein (VP1/VP2), respectively [1]. VP1 and VP2 are identical except that VP2 has a shorter N terminus [1]. Parvoviruses infect a wide range of vertebrates and invertebrates and many members are associated with clinical disease such as reproductive failure, respiratory disease, enteritis, panleukopenia, hepatitis, erythrocyte aplasia, immune complex-mediated vasculitis, and cerebellar ataxia [2–5]. To date, three subfamilies, *Parvovirinae*, *Densovirinae* and *Hamaparvovirinae*, have been proposed in *Parvoviridae* [6]. Eleven genera have recently been defined in subfamily *Parvovirinae* by ICTV (https://ictv.global/taxonomy), including *Tetraparvovirus*. The first Tetraparvovirus ungulate 1 were identified in 2007–2008 from domestic cattle from local food markets in Hong Kong, which was named as bovine hokovirus and showed a close relationship to human parvovirus 4 (now named as Tetraparvovirus primate 1), with 62.9–63% genomic identities to the human parvovirus 4 [7, 8]. Later in 2016 this virus has been reported in apparently healthy domestic yaks (*Bos grunniens*) from Guansu and Qinghai provinces of China [9]. In addition to the cattle in HongKong and domestic yaks from northwestern China, Tetraparvovirus ungulate 1 was lately detected in 2016 for the first time from asymptomatic and sick steers from Mexico and USA [10] and from cattle in the South of Brazil [11]. Another Tetraparvovirus ungulate 1 strain (OP113956) was identified from Ireland upon BLAST search in GenBank. Hitherto, the Tetraparvovirus ungulate 1 has been discovered in Asia, North America, Brazil and Europe, which may suggest it presents in cattle populations throughout the world, however, most of the reported Tetraparvovirus ungulate 1 were identified from apparently healthy or asymptomatic individuals, and the information including the virus genetic variation and its pathogenicity is still very limited.

In the present study, we identified and characterized a Tetraparvovirus ungulate 1 strain from diseased Chinese Simmental in a cattle farm from central China. Based on the genetic analysis of the known genomes of Tetraparvovirus ungulate 1, two distinct genotypes (I and ΙΙ) could be defined, and the present Tetraparvovirus ungulate 1 was clustered into genotype II. This is the first report of Tetraparvovirus ungulate 1 in the domestic cattle from mainland China.

## Samples collection

In October 2023, during the routine testing, five serum samples of Chinese Simmental were sent to our lab by the local veterinarian for detecting possible causative viral pathogens. The animals were from a free-range cattle farm located in Chengbu county of Hunan province, China, and many individuals around 210-day-old were suffering from emaciation, jaundice, sudden weakness in limbs, inability to stand on the ground, and subsequent death, with no response to antibiotic therapy by the local veterinarian. The collected serum samples were stored at -80 °C until use.

## Virus detection

One hundred microliter (µl) of the sera from each sample was used for viral nucleic acid isolation according to the protocols of the DNA/RNA extraction kit (Axygen). The viral RNA was reverse transcribed to cDNA using a RevertAid First Strand cDNA Synthesis Kit (Thermo Scientific) according to the manufacturer’s instructions. The presence of some possible pathogens, including circovirus and astrovirus, were investigated by PCR with degenerate primers as described previously [12, 13]. Peste des petits ruminants virus, bovine viral diarrhea virus and bovine enterovirus were also checked by PCR with primers designed by the present study (Table S1). However, no positive sample was detected by the PCRs with the present primers, so an sequence-independent single primer amplification (SISPA) were used to detected possible virus in the DNA extracted from 200 µl sample pooled by the five sera with 40 µl each, as described previously [14, 15], and the amplified products were sent to Tsingke Biotech (Beijing) for library preparation and sequencing using an Illumina HiSeq Х10 platform. The clean data were used to perform a BLAST search. The results showed that most of the reads had high homologies to Tetraparvovirus ungulate 1 (bovine hokovirus), and then they were assembled into several contigs, while there were many ambiguous nucleotides within the contigs. In order to get the authentic genome, based on the obtained contigs and the conservative region of the known Tetraparvovirus ungulate 1, four pairs of primers (Table S1) were designed to re-amplify the nearly complete genome of Tetraparvovirus ungulate 1, and the primers ChBHkV-22 F/631R were used to re-check Tetraparvovirus ungulate 1 in the samples. All of the five sera were PCR positive by the primers ChBHkV-22 F/631R, and one sample (number 1) was chosen to re-amplify successfully the nearly complete genome of Tetraparvovirus ungulate 1, which was designed as HNU-CBY-2023.

## Genomic characterization

The obtained genome of HNU-CBY-2023 was 5346 nt in size, and showed identities of 85-95.5% to the known Tetraparvovirus ungulate 1 from GenBank (Table 1, Table S2), indicating a rather genetic divergence with other known Tetraparvovirus ungulate 1. Three ORFs similar to other tetraparvoviruses were also predicted, including *NS1*, *VP1* and *VP2* genes, which encoded proteins of 652 amino acids (aa), 931 aa and 553 aa, respectively. Four motifs (A, B,Bˊ and C) of superfamily 3 (SF3) helicase and the conservative amino acids within putative NTP binding motif [16, 17] were identified in NS1 of HNU-CBY-2023 (Fig. S1).Moreover, the conserved motifs of the Ca^2+^ binding loop (YXGXG) and the catalytic center (HDXXY) of phospholipase A_2_ (PLA_2_) were also found in VP1 (ORF2) of HNU-CBY-2023 (Fig. S2), which was reported to play an important role in entering the host cell and be required for infectivity of parvoviruses [18, 19].

**Table 1:** Identities (%) between the present Tetraparvovirus ungulate 1 strain HNU-CBY- 2023 and other Tetraparvovirus ungulate 1 strains from genotype I and II

	Genome		NS1 nt*/amino acid		VP1 nt/amino acid	
	Genotype I	Genotype II	Genotype I	Genotype II	Genotype I	Genotype II
HNU-CBY- 2023	85-86.5	92.7–95.5	88.1–90.2/92.2–96.6	96.9–97.8/99.5–99.7	89.5–90.6/94.3–97.7	96.6–97.9/99.7–99.8

*nt: nucleotide

## Phylogenetic and recombination analysis

**Fig. 1:**
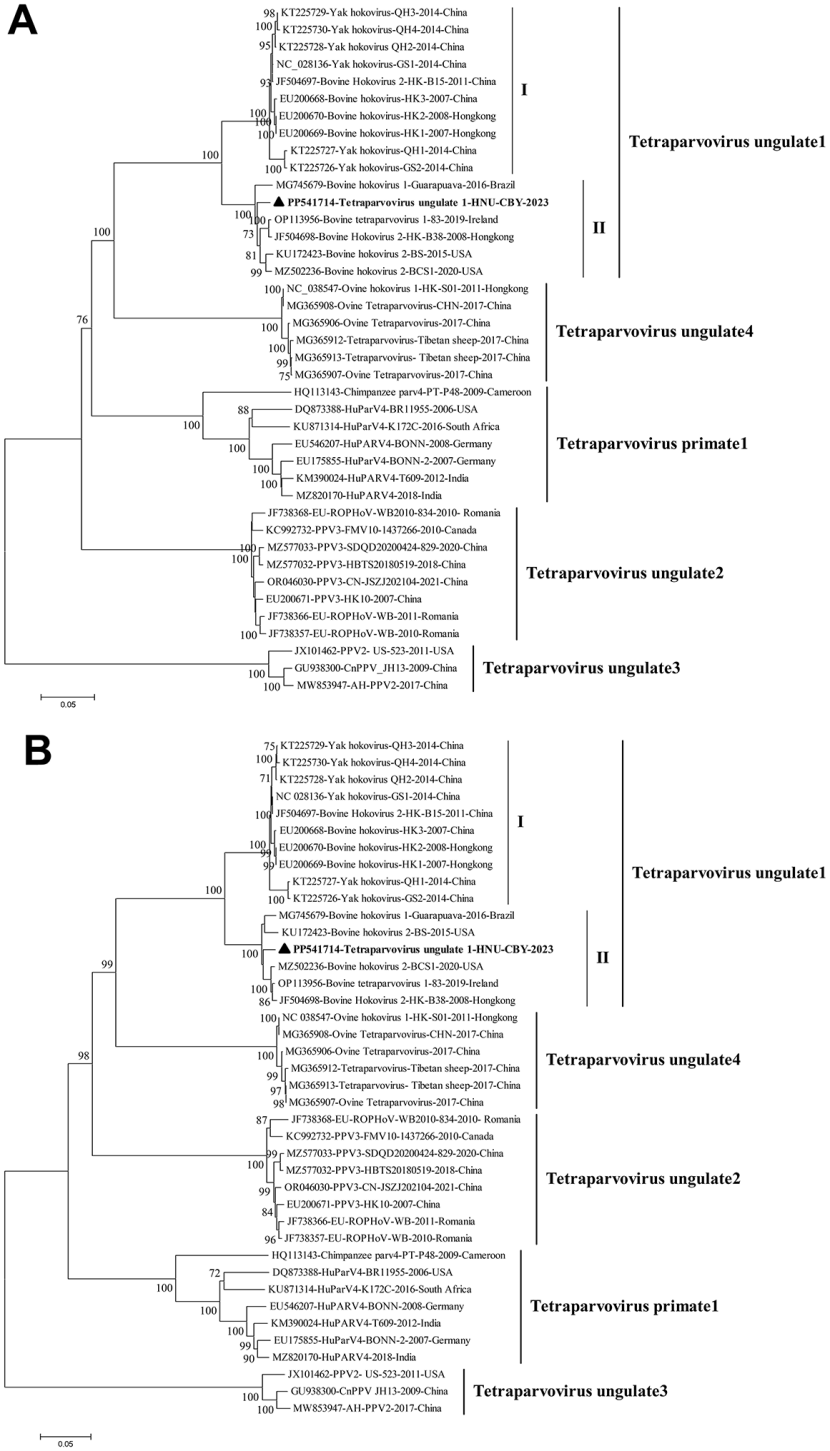
The phylogenetic trees were constructed by using the Neighbor-Joining method based on *p*-distance model, with the nucleotide sequences of the genomes and nonstructural protein genes (*NS1*) of the present Tetraparvovirus ungulate 1 and other represent parvoviruses within genus *Tetraparvovirus*. The percentage of replicate trees in which the associated taxa clustered together in the bootstrap test (1000 replicates) are shown next to the branches (only > 70% are shown). The trees are drawn to scale, with branch lengths measured in the number of substitutions per site. The analyses involved 40 sequences of parvoviruses with their GenBank accession numbers indicated. Tetraparvovirus ungulate 1 strain HNU-CBY-2023 characterized in the present study are in bold and marked with “▲”. (**A**) Tree constructed based on the genomes. All positions containing gaps and missing data were eliminated. There were a total of 4455 positions in the final dataset. (**B**) Tree constructed based on the nucleotide sequences of the *NS1* gene. All positions containing gaps and missing data were eliminated. There were a total of 1884 positions in the final dataset. Evolutionary analyses were conducted in MEGA7 [20]

Further phylogenetic analysis based on the nucleotide sequences of the genomes, *NS1* and *VP1* genes were performed, including the present Tetraparvovirus ungulate 1 strain HNU-CBY-2023, other Tetraparvovirus ungulate 1 strains and some other representative tetraparvoviruses from GenBank (Fig. 1 and Fig. S3). The phylogenetic results consistently showed that Tetraparvovirus ungulate 1 strains were clustered into two distinct clades, with a very high bootstrap support of 100% (Fig. 1 and Fig. S3), indicating Tetraparvovirus ungulate 1 could be divided into two genotypes (I and II), which was further supported by a rather high average genetic distance of 0.102 ± 0.004 between the genomes of the strains from genotype I and II as calculated by MEGA7 [20]. The present strain HNU-CBY-2023 was clustered into genotype II, and showed a substantial genetic divergence to the strains from genotype I (Fig. 1; Table 1). Interestingly, it seemed that the strains from genotype II (from 2015 to 2023) is younger than those from genotype I (from 2007 to 2014) (Fig. 1), which may indicate that a genotype shifting from I to II is occurring as happened in PCV2 [21], however, investigations with more samples from different regions are needed to verify it. Furthermore, no recombinant event was detected in HNU-CBY-2023 by software RDP4 [22].

## Discussion

Most of the published Tetraparvovirus ungulate 1 strains were discovered from animals with no obvious symptom or from food markets or from commercial bovine serum [7–9, 23]. One study carried on 2015 reported that Tetraparvovirus ungulate 1 could be detected in asymptomatic cattle in Mexico with a positive rate of 3.8%, and could be found both in sick (25%) and asymptomatic (5%) cattle from USA [10]. Interestingly, the first tetraparvovirus was identified in 2005 from the plasma sample of a homeless, daily injection drug user who presented with an acute viral infection, including fatigue, vomiting, diarrhea, sore throat, neck stiffness and joint pains, and coinfected with hepatitis B virus [24]. The viremia of this virus was found in 2% (4 out of 200) of plasma samples from healthy blood donors and in 6% (13 out of 216) of plasma samples obtained from febrile patients with symptoms resembling acute human immunodeficiency virus (HIV) infection [25]. Human PARV4 DNA was also reported to be persistent in tissue from some HIV-infected individuals, but was not identified in tissue from a small number of subjects not infected with HIV [26], while another report indicated that PARV4 DNA persisted in liver tissue of a wide range of individuals, and the increased prevalence of PARV4 in HIV, HCV, IVDUs (intravenous drug users) and other high risk groups may be due to increased exposure to infection, but persistence was clearly not exclusive to these groups [27]. Moreover, Human PARV4 was found to be associated with acute encephalitis syndrome [28, 29] and severe acute respiratory infection (SARI) [30]. In general, these studies on human tetraparvovirus (classified as Tetraparvovirus primate1) indicated it may play a role in human clinical disease.

## Conclusions

In the present study, we identified and characterized the genome of Tetraparvovirus ungulate 1 from Chinese Simmental suffering from severe disease, and compared it with other published sequences of Tetraparvovirus ungulate 1 strains from GenBank. The results demonstrated that Tetraparvovirus ungulate 1 could be phylogenetically divided into genotypes I and genotype II, with strains in genotype II identified more recently, which may indicate a genotype shifting from I to II in the field and further surveillance could be necessary.

This study is the first report of Tetraparvovirus ungulate 1 in domestic cattle from mainland China, which will help to understand the prevalence and genetic diversity of Tetraparvovirus ungulate 1 in China and abroad. However, till now, there is no report on the successful isolation of tetraparvovirus from animals or human beings, which makes it very difficult to investigate precisely the pathogenicity of tetraparvovirus. Further researches, including finding appropriate cell lines and conditions to isolate, amplify this virus and also animal experiments, are essential to clarify the pathogenicity of tetraparvovirus to animals and humans.

### Electronic supplementary material

Below is the link to the electronic supplementary material.

Supplementary Material 1

## Data Availability

The sequence described in this study has been deposited in the GenBank database under accession number PP541714.

## Abbreviations

aa: amino acids
HIV: human immunodeficiency virus
HCV: hepatitis C virus
IVDUs: intravenous drug users
NS1: nonstructural protein
ORF: open reading frame
PCR: polymerase chain reaction
PCV2: porcine circovirus 2
PLA_2_: phospholipase A_2_
SARI: severe acute respiratory infection
SF3: superfamily 3
SISPA: sequence-independent single primer amplification
VP1: virus particle protein 1
VP2: virus particle protein 2
