# Supplementary material for: First detection of Tetraparvovirus ungulate 1 in diseased cattle (Chinese Simmental) from Hunan province, China
Source: Virol J. 2024 Jun 6;21:132. doi: 10.1186/s12985-024-02402-1 (PMC11155090; doi:10.1186/s12985-024-02402-1)
Supplement: Supplementary file 1 — Supplementary Material 1 [file 12985_2024_2402_MOESM1_ESM.docx]

**Supplementary Information**

**Table S1** Primers designed in the present study

| Primer | Sequences（5‘ to 3’） | Size (bp) | Reference |
| --- | --- | --- | --- |
| BVDV-DF1 | CTCGAGATGCCAYGTGGACGAG | 161 | This study |
| BVDV-DR1 | CCATGTGCCATGTACAGCAGAG |  | This study |
| BoETV-2206F | GAAGCAATGCTGGGCACCCAC | 460 | This study |
| BoETV-2666R | CACCACGTTTCGAGTCTCTATGAG |  | This study |
| PPRV-DF | CAGCCTATGAGAGCGGAGTAAGAATC | 974 | This study |
| PPRV-DR | GTGGTGTCCAACATCCCGGCTAT |  | This study |
| ChBHkV-22F | TTCTTTTGAGCGAGGTGGGTGTGACGT | 610 | This study |
| ChBHkV-631R | GAAGGTGCGCCATCTCTTTAGCCAAG |  | This study |
| ChBHkV-414F | 5-GCCACTTTGATCTAGCAGTTAACTTAG | 1625 | This study |
| ChBHkV-2038R | CCACCAGCTCTCTATCAGAGGGT |  | This study |
| ChBHkV-3300F | CTATAGGTGATGTGGCAGATGTTCAAAT | 1513 | This study |
| ChBHkV-4812R | GGGTACTGAGCAAAGAAGTGACATTC |  | This study |
| ChBHkV-4663F | GACCGAGACAGAGTTCAATCCTAAACT | 720 | This study |
| ChBHkV-5383R | TTTGAGCGAGGTGGGTGTGACGTC |  | This study |

BVDV: Bovine viral diarrhea virus; BoETV: Bovine enterovirus; PPRV: Peste des petits ruminants virus. ChBHkV: the bovine tetrapavovirus identified in the present study.

**Table S2** Identities (%) between the present Tetraparvovirus ungulate 1 strain PP541714-HNU-CBY-2023 and other Tetraparvovirus ungulate 1 strains.

| **Genotype** | **Strains** | **Genome** | **NS1 nt*/amino acid** | **VP1 nt/amino acid** |
| --- | --- | --- | --- | --- |
| II | OP113956- Ungulate tetraparvovirus 1-2019-Ireland | 95.5 | 97.8/99.7 | 97.8/99.7 |
|  | JF504698-Bovine Hokovirus 2-HK-B38-2008-Hongkong | 95.5 | 97.5/99.5 | 97.9/99.8 |
|  | MG745679-Bovine hokovirus 1-Guarapuava-2016-Brazil | 95.5 | 97.1/99.5 | 96.6/99.8 |
|  | KU172423-Bovine hokovirus 2-BS-2015-USA | 94.9 | 96.9/99.5 | 97.3/99.8 |
|  | MZ502236-Bovine hokovirus 2-BCS1-2020-USA | 92.7 | 97.4/99.7 | 97.2/99.8 |
| I | EU200670-Bovine hokovirus-HK2-2008-Hongkong | 86.5 | 90.1/96.6 | 90.6/97.7 |
|  | NC_028136-Yak hokovirus-GS1-2014-China | 86.5 | 90.1/96.5 | 90.6/97.5 |
|  | EU200669-Bovine hokovirus-HK1-2007-Hongkong | 86.4 | 90.1/96.6 | 90.6/97.7 |
|  | JF504697-Bovine Hokovirus 2-HK-B15-2011-China | 86.3 | 90/96.5 | 90.6/98.2 |
|  | EU200668-Bovine hokovirus-HK3-2007-China | 86.1 | 90.1/96.5 | 90.5/97.7 |
|  | KT225728-Yak hokovirus QH2-2014-China | 86.3 | 89.6/95.7 | 90.5/97.3 |
|  | KT225729-Yak hokovirus-QH3-2014-China | 86.1 | 89.4/95.3 | 90.4/96.9 |
|  | KT225730-Yak hokovirus-QH4-2014-China | 86.0 | 89.2/94.8 | 90.2/96.7 |
|  | KT225727-Yak hokovirus-QH1-2014-China | 85.0 | 88.2/92.5 | 89.5/94.3 |
|  | KT225726-Yak hokovirus-GS2-2014-China | 85.0 | 88.1/92.2 | 89.5/94.5 |

*nt: nucleotide.

CPP541714-HNU-CBY-2023 MDATCFTAVLQVPTVFCEAVPVQFQQPAAVLREVPDEYPDWPVSDLRQYASHFDLAVNLGHILYRELLESFAMILPLYPQ 80

EU200668-BOVINE_HOKOVIRUS-HK3 -----------------D---------SV------------------------------------------S-------- 80

JF504698-BOVINE_HOKOVIRUS_2-HK-B38 ----------------------------T--------------------------------------------------- 80

NC_007018-HUMAN_PARV4 ---PAWI----I--G-LSN.-ANWRDWDGLQ-PRNLLAD---IQE--ESVPF--H-----YCILQQ-FA-H-VT--CRVK 79

JQ425257-PPV3-US-HK238 -E-S------H--KSL--SAA-L-EY--DI-TS---VIE-------QSASEY--IG----R-I-------------QHRS 80

JX101462-PPV2-US-523 -AGLMYLG--KLYRGDL-P..-RWL-GDRMANRNMPAPE---IEQ-CDKWGREMFRFLVSVFELIDETLQVTYDNYFRER 78

AF406966-BPV2 ..MSYY-F-VTI-DKIEDYG.QAYYDVVNRVTLTHRDNEK-VYDPEDREIIFKQAQ-YVDCLER.....RLKQM-VSGSM 72

CPP541714-HNU-CBY-2023 PKLFLQLEPSK.SDDREFHYHLVCEQGQMSG.REFSTWLKRWRTFMDRYMAGDHVWGLLWNIRKTRQGRLYQADLSFVVR 158

EU200668-BOVINE_HOKOVIRUS-HK3 -----------.-------------------.--------------------H--------------------------- 158

JF504698-BOVINE_HOKOVIRUS_2-HK-B38 -----------.-------------------.------------------------------------------------ 158

NC_007018-HUMAN_PARV4 -SM-------S.GEEN-M-----VN-AD-V-.--C-N--RT-KV--AG-LVAP.--T-S----E----------M---KN 156

JQ425257-PPV3-US-HK238 ----------S.-EEKA------A---NV--.----N-----A--LT-H--PG-S-SI--D-----------C--T--L- 158

JX101462-PPV2-US-523 I-H---M---SPGNPAP--L--AVSPPRHK-P--MGG---MF-QA-IEHRP-T.APP-YFEM---AH--WWEG-QD-IRC 157

AF406966-BPV2 YNY-I---QGE..KHKRW-I-V-LDISVGNP.-NCREVIEKVEYEYNSICY-R..PVRQTQ-NR-SN-SWKIETED-IIN 147

CPP541714-HNU-CBY-2023 YLLPKLPVENCWFAWSNIERFQASLLSVPNRVRCLGEGGAIALPYVEGAVALPEGHAVSEAPSMGGKGTDRFLSLIDWLV 238

EU200668-BOVINE_HOKOVIRUS-HK3 ------------------D----------------------------------G-Q--L--------------------- 238

JF504698-BOVINE_HOKOVIRUS_2-HK-B38 -------------------------------------------------------------------------------- 238

NC_007018-HUMAN_PARV4 -------LND-YY--T--D--E-AV---R--Q.LS-PQ------FTDAPPRT-AAEG..VP-T-A----Q--MD------ 233

JQ425257-PPV3-US-HK238 -----V---HV-Y--T-MD--E-AA----A-----S--------FTG-SGEASGS...-S--T-A----E---N------ 235

JX101462-PPV2-US-523 --V----PGDVIW-STSMQE-DEVV--E-K-R...DYA-PLP-GDGSQDPRQGGQEE.HPG-TVK-RAA----QAV---- 233

AF406966-BPV2 ---C-I-PKEAKY--T--KNKIG..............DACLNIDLRKEISVR-DID....V-MWRQASG--MRD-VE-CI 209

CPP541714-HNU-CBY-2023 EQGIATERRWLNADKRSYRSFLGSSGGVLQARNALLVARREMVLAHPLLGYLQ...........RGGSAVAA..DNRVVE 305

EU200668-BOVINE_HOKOVIRUS-HK3 -----------S-----------------------------------------...........--------..----I- 305

JF504698-BOVINE_HOKOVIRUS_2-HK-B38 -----------------------------------------------------...........--------..------ 305

NC_007018-HUMAN_PARV4 -N-----K---SVN-L---------------T---QI-K----------S--T...........KNA--FEE..S-K-AQ 300

JQ425257-PPV3-US-HK238 -E---------AT--K-------------------Q---------R------R...........----DI-S..--K-T- 302

JX101462-PPV2-US-523 -N-CC--AK-IEM--MG----MST-Q----VK---NL----L-AGNK--ESIV...........K-ADPWLPGVV-A-AR 302

AF406966-BPV2 KNHVF--DQYMAKFEE--Y--ACTNQ-RHMLQTS-EL-AKRTTSMI--GV--AGFSNIQEAIEEANKTEF-DIYN-K-FD 289

**A**  **B**

CPP541714-HNU-CBY-2023 LFKINGYDPVDAAWYFAAWAQGIWPKRRALWLW**G**PAST**GK**TLLAAAIANISPSYGCVNWTNQNFPFNDCHCQSLV.WWEE 384

EU200668-BOVINE_HOKOVIRUS-HK3 ---L-A---E-----------------------------------------------------------------.---- 384

JF504698-BOVINE_HOKOVIRUS_2-HK-B38 -------------------------------------------------T-------------------------.---- 384

NC_007018-HUMAN_PARV4 --SL---N------------R-V------I-------------------L-------------------------.---- 379

JQ425257-PPV3-US-HK238 --R------E----------A-A-A----M------------------AVA------------------------.---- 381

JX101462-PPV2-US-523 --H------EQ----L-N--K-GHQ-------F---N-------G-F-KLA-CF-----N-E----S--AS---I.---- 381

AF406966-BPV2 -LQFQ-----V-GYIIY--SIRATGR-G---FY--GQ---SIM-R-M-TC-VR--------S----Q-LATNCQIG---- 369

B**ˊ**  **C**

CPP541714-HNU-CBY-2023 GRMTENIVEVA**K**AILG**G**APVRLDVKNKGSEDFIPTCVIITSNGDLTVTVDGPVVSTVHQEALQTRITMFQFNRLVPPG.. 462

EU200668-BOVINE_HOKOVIRUS-HK3 ------------------------------------------------------------------------------.. 462

JF504698-BOVINE_HOKOVIRUS_2-HK-B38 ------------------------------------------------------------------------------.. 462

NC_007018-HUMAN_PARV4 -------------V-----------------Y------------------------Q--------------Q-M--D-.. 457

JQ425257-PPV3-US-HK238 --------------------------------------------------------A--------------Q------.. 459

JX101462-PPV2-US-523 -K-S-KF--A-------SGI-I-I-G-P--Q---AP-V------MCTVYS-N-I--A-AGP-KS-MLKVT-SHVL-G-PN 461

AF406966-BPV2 -VI--D---S---L-S-GKI-V-R-CRD-VEIT-PPFV----N-M-LVQG-NQ--F--KKP-ED-MIK-N--KRL--N.. 447

CPP541714-HNU-CBY-2023 ..LAPIPEADLHDFFKWGSELLKDKGSPPEAFRVP....RRFDKPTLSDVSRSP...RLPIRDAIESRESWSSEDEWFPS 533

EU200668-BOVINE_HOKOVIRUS-HK3 ..---------------------------------....---------------...------V---------------- 533

JF504698-BOVINE_HOKOVIRUS_2-HK-B38 ..---------------------------------....---------------...----------------------- 533

NC_007018-HUMAN_PARV4 ..---L--EEVRS---L-EQE-NM--T---E----....-N---QPMAST-NL-...KALCAPMEDNQVQ-D---D---P 528

JQ425257-PPV3-US-HK238 ..---L--R--R---A--AQ--ES--A---Q----....--S---R--EII---...----..-A-E-PT--------TP 528

JX101462-PPV2-US-523 AD-P-WVLR--PS-MAY-QK--NER-T---IDQLA....VAAAAAPCQVSTTA-KIE-SALPPIRAG-DT-D--E----D 537

AF406966-BPV2 ..FGIVERE-MKQ----S-FIYYN-LL-K-QAYLSDPKLIGHVV-YT-FLRQTAKVEKIVRPFEEQEQ-DLAEL-S--AD 525

CPP541714-HNU-CBY-2023 .....SRSSASIRPGT.......SAPQSSPAASPHTPDTAAALASGPFPEADSDEERYAHASDD....PLIESWWCQFLA 597

EU200668-BOVINE_HOKOVIRUS-HK3 .....----SA---R-.......-T---------P------------------------R-A--....---------Y-- 597

JF504698-BOVINE_HOKOVIRUS_2-HK-B38 .....----S------.......-----------------------------------------....------------ 597

NC_007018-HUMAN_PARV4 PTQKKR-ELQETP-T-PSEVIEL-S-SPLAD-P-R---SLGE-SLT-TSVSQIVSAPFPDETAERYGAGD---F-SEHVF 608

JQ425257-PPV3-US-HK238 .....N-E....--..........TTPEV---DES-DVSVSE-V-L----L-.ES---QRDFPL....DVDAA-LDV-V. 583

JX101462-PPV2-US-523 P....PQNTPTRM-RE.......RQKTRRLTEYSDSETDVEEEPRYGA-AREALGYLFLPECASRVTRRHQPGKTL-RIL 606

AF406966-BPV2 ..........................PPYMGKP-KKSE.......................................... 537

CPP541714-HNU-CBY-2023 DFDWAERLYLVGPQGPRARGLFYYQLWIREPWRYRRTLRHSESALKSKLYSWWNW. 652

EU200668-BOVINE_HOKOVIRUS-HK3 -------------------------------------------------------. 652

JF504698-BOVINE_HOKOVIRUS_2-HK-B38 -------------------------------------------------------. 652

NC_007018-HUMAN_PARV4 -A---T--HICP-G--KPY---WTY--S--F--FKQS-SR--AH-INRRFI-AW-. 663

JQ425257-PPV3-US-HK238 ..----N-N-RA-GA---GS-YR-HQ-LS------Q--SRT-GYVR-RMHD-F--. 636

JX101462-PPV2-US-523 RSWD-R-SSVARSEAACT-ARHDVDN-YYHIP.CLSLIGG-VRPSNTV-RP-QA-L 661

AF406966-BPV2 ........................................................ 537

**Fig. S1** Amino acid sequence alignment of NS1 of the present bovine tetraparvovirus HNU-CBY-2023 and other represent parvoviruses, with the putative motifs of superfamily 3 (SF3) helicases. A, B, Bˊ and C above the sequences indicate the putative helicase motifs. Boldface letters represent amino acids which are invariant within the parvovirus nonstructural proteins examined. Underlined boldface letters represent amino acids which are invariant within the superfamily of small virus putative NTP binding proteins. PPV: porcine parvovirus; BPV: bovine parvovirus.

**CPP541714-HNU-CBY-2023**  MSAADAYRPGDRLPLDTLMSKIT..YDVGFEPTRHGGRGGGGYLGELTTGLLFTKAFHELSRYASNLP...YELFPIKQL 75

EU200668-Bovine_hokovirus-HK3 -----------------------..-------------------------------------------...------R-- 75

JF504698-Bovine_hokovirus_2-HK-B38 -----------------------..-------------------------------------------...--------- 75

NC_007018-Human_PARV4 ----------GK----E--QRMNRAIP--P--SSQAN----P-QTHFAI-IMYS---QG-L-F-NA--...P--S-V--- 77

JQ425257-PPV3-US-HK238 -T----FK--QPF------E-L-..P---P--SSWSR-M-----AS-G---V-KQ--SHVDKFIRK--...AKVY-V--F 75

JX101462-PPV2-US-523 ------WK----P--EN--DAMDR.SIW-DRRNNS---R-INA-LRAGYDPRMR--LLQ-YKFFISIKKQGGFWDK--EV 79

AF406966-BPV2 ....M-AKL-GG-VGEAAIHR-ES..LS-KVHYKDKYWFPDN-VYTTGNQEEED-IVAK-LLDPNAPVKEWGNGRWSFEY 74

CPP541714-HNU-CBY-2023 VNQLINKRRETNNIHKVMRFFMDVIRLLLTVSHGPAEQRLRQAMAPVADFFYEPSRHQFAIGPNVSPLQDLARYAHTAQK 155

EU200668-Bovine_hokovirus-HK3 -------------------------------------------------------------------------------- 155

JF504698-Bovine_hokovirus_2-HK-B38 -------------------------------------------------------------------------------- 155

NC_007018-Human_PARV4 ----E-Y--K-SDTRVWY-VYL-MT---IS-APPG-ANK----A-GIT..........HSKA--AES-RGIV-F-AA-FV 147

JQ425257-PPV3-US-HK238 AQ--M-YW-KSTD-KTIG-----LV---IL-APE--KAK---SALNLG.........D-HRP--PAV-L--HKF-KD-VV 146

JX101462-PPV2-US-523 AK-WWMAGHQ-SKGDELA-TVL-LG--FMA-VDDYR-DPEIDSVRRSMID......FLYGDNDATRAFFG-Q-F-DKVHL 153

AF406966-BPV2 GPEWRRLGFIPHTDDINY-GLSYHKAPGTQLLE-IH-IPPE-KQG-ESIT.......-I-PPTQD-V-LEPMTNDPHYK- 147

CPP541714-HNU-CBY-2023 PTREAIEAFFQGMLSPIKPEDR.......DILDTIQKQFEEFFYPPIVEHAGPGDYSRDGDTGAGTPSESD...LERPAN 225

EU200668-Bovine_hokovirus-HK3 ----------------------.......P-----------------------------------------...------ 225

JF504698-Bovine_hokovirus_2-HK-B38 ----------------------.......------------------------------------------...------ 225

NC_007018-Human_PARV4 --V-N-DR--EDS-TNFAK--L.......-TWQQLHE--IKL-H--D-GVHLVS-.--..-E--DSLV-P-...-----G 214

JQ425257-PPV3-US-HK238 -SA-D-KI--EESV--WSE--K.......K--KQ-EG--Q-I-H--TDTED-ADS-GGNS-Q-T-SL--P-...----PE 216

JX101462-PPV2-US-523 -Q-DVMRVWIT-GYR-QP-KGSP.WGTWAELDLD-RNSLDASYKAEEEDRSKAIQVP-IN-P-SESGEQPQPAPE----E 232

AF406966-BPV2 -KPPGDLGDIPKP-T-WQLANKIGPDYARQL-TMSGHD-TGRTP-VQIPDP-IREGLKELEEQSKLMERVQAGEGDEKPR 227

■ ■ ■ ●● ●

CPP541714-HNU-CBY-2023 R...........................................GLLVPGYN**Y**V**GPG**NPLDNGPPQGPVDEAAKH**HD**ER**Y** 262

EU200668-Bovine_hokovirus-HK3 -...........................................------------------------------------ 262

JF504698-Bovine_hokovirus_2-HK-B38 -...........................................------------------------------------ 262

NC_007018-Human_PARV4 G...........................................--TL-------------S------------------ 251

JQ425257-PPV3-US-HK238 -P..........................................-I----------------A-AK-------------- 254

JX101462-PPV2-US-523 SAGAAGPGSDGQSGRADTGAGRERDSPSTEVGGSDGPESDGKGG--TL---R---------A-E-R----AI--K----- 312

AF406966-BPV2 G...........................................--TL-KHR-----GD-PA-R-MSKL--I-AR--IG- 264

●

CPP541714-HNU-CBY-2023 DEMLSHGDLPYVHGHGA**D**RLMNKEIERAEAEGRVTGVGDKLLGNVIRGIWEAKETIGDVADVQISQVLP.......PAPP 335

EU200668-Bovine_hokovirus-HK3 -----------------------------K---------------------------------------.......---- 335

JF504698-Bovine_hokovirus_2-HK-B38 ---------------------------------------------------------------------.......---- 335

NC_007018-Human_PARV4 A--IE---I--L------------L-EK-RR-DI-HLA-VVV--A---L-Q----V--I----L-----.......---- 324

JQ425257-PPV3-US-HK238 ----R------I--R--------------Q--KIDNPV-A-V--A----------L--I----L-----.......-D-- 327

JX101462-PPV2-US-523 --LIK--HI--I--R---S--G--LAD--EA-KILDRY-Q-VA-AA--L-R--D-LA-LIGGELDN---.......-D-- 385

AF406966-BPV2 HTEIK-KHN--YWYNFY-EQ-V---N..-NMDQIAEE-ESW-A-F-LAT-K--ASFTNPLGILLEHLQ-DWATYYD--NT 342

CPP541714-HNU-CBY-2023 PQVP................RADSSDGLPS.......AKRARPGIPEDPVAADFDIASPDP................... 373

EU200668-Bovine_hokovirus-HK3 ----................GV--------.......S-------------T---L-----................... 373

JF504698-Bovine_hokovirus_2-HK-B38 ----................----------.......------------------------................... 373

NC_007018-Human_PARV4 SSDQ................QPAY-A-E--.......--K--I-T-DESDP-LLLQPHTN.................... 361

JQ425257-PPV3-US-HK238 TSQV................LPG--EDA--.......P--Q-A-T-DSVPTPGNPSPA-LA................... 365

JX101462-PPV2-US-523 V-SEGEESQKRPREEEDPPES-N-APQV-P.......-QKP-LDV--YFWTDEEEG-GEGSGDDEGGGIVRINI...... 452

AF406966-BPV2 HHKQWIAFQRALTQHGTASTERPRTPPT-DNEMFPHL---P-LDE-NKSASCST-MS-CEISSTQCTNDEDMELNQTTVM 422

CPP541714-HNU-CBY-2023 ............LAAPEMASEATGGAGGGIKVKAQWIGGTSFTDSLILTAHTRTSMLADRGGYVPVYRKGSHTDE.SQPV 440

EU200668-Bovine_hokovirus-HK3 ............IV--D----------------------------------------------------------.---- 440

JF504698-Bovine_hokovirus_2-HK-B38 ............---------------------------------------------------------------.---- 440

NC_007018-Human_PARV4 ................T-SV-PA--.---V------------S--VVI-S-----------------KQ---V-S.---- 423

JQ425257-PPV3-US-HK238 ............DP-TI--APV--AT---V------L---H-S-NT-V-S------------------S---VSD.R--- 432

JX101462-PPV2-US-523 .........PIKMQSADSTQHQPQ-H---PGASGH-RA--V-GTHGVT-TQ--MVI-SSKTD-K-LFLDADTSKFD-E-G 523

AF406966-BPV2 GMPSSTTDSTIGNTGIQGTGDCA--G--AQRCTNK-L--I-W-HNTFT-YQ--RCI-QP...FTNK-TFT-SV-S..T-G 497

CPP541714-HNU-CBY-2023 MGMRTPYSYIDVNAISAHLTPRDFQQLLDEYEEIRPKKLIVGISAIVIKDVATNSTGT....TVSDSASGGITIFSDDSY 516

EU200668-Bovine_hokovirus-HK3 -----------------------------------------------------T----....----------------G- 516

JF504698-Bovine_hokovirus_2-HK-B38 ----------------------------------------------------------....------------------ 516

NC_007018-Human_PARV4 ---K----------L---F------------D--K--S-TIA------------Q---....-----------V-A---- 499

JQ425257-PPV3-US-HK238 ---------------------------------------VI---G------SVTT---....-----------V----A- 508

JX101462-PPV2-US-523 --FL--WQ-F-F-CYMN-F--S-W-E-GRR-DS----S-TISVENV-----HQTNNE-....N-H--GT---M--E-SE- 599

AF406966-BPV2 ITVT--WY---L-CFYS-IP-STM-EIIETTDGFK-LT-T-T-TE--G---SCTT-SSGVPN--T--QTATLLLHR--H- 577

CPP541714-HNU-CBY-2023 EYPYVLGHNQDTLPGHLPGENYVLPQYGYLTRGREIDSSTSH...............DIVGIADHKTELYFLEHHDAQCL 581

EU200668-Bovine_hokovirus-HK3 ---------------------------------------S--...............----------------------- 581

JF504698-Bovine_hokovirus_2-HK-B38 ------------------------------------------...............----------------------- 581

NC_007018-Human_PARV4 D----------------------------I-------QQN-.................--A-S------F-------E-- 562

JQ425257-PPV3-US-HK238 D----------------------------------F-KG-D.................-------RS----------E-- 571

JX101462-PPV2-US-523 TF---I--A-EGN--A-SIQW-NP---A-F-GFNP-AWGHANGT............TKYRVHPSAN--FFV--E-A--I- 667

AF406966-BPV2 -L---I-GG-E-V-E----DW-K----C-T-V-M-SPWGNWSQARPDDCAGNTWSVNCTNYYSTQDS--FL--NLVNTQ- 657

CPP541714-HNU-CBY-2023 GSGDTWSHVYDFPKDLPFRRLSTPSQTLYGRHNPIPSSRLAIMTGVDSQGKAVWKRPTGMDVG..RLPLNHVPGPALMTP 659

EU200668-Bovine_hokovirus-HK3 -------------T-------------------------------------------------..--------------- 659

JF504698-Bovine_hokovirus_2-HK-B38 ---------------------------------------------------------------..--------------- 659

NC_007018-Human_PARV4 -T--H---H-E--D---W-K----N----A-----------------ND-T-I----E-----..-----Y-------M- 640

JQ425257-PPV3-US-HK238 --------A-E--S-------T--N-S--A------P----------TS-VPK----A-E---..KH---Y----SV-M- 649

JX101462-PPV2-US-523 R---GT-FA-E--.S-DPK--GSRMG--NL----VLP-----YL-Q-GSDAPTFYQ-Q-T-LD..IF-QGFI---RPCL- 744

AF406966-BPV2 HP-CS-TSQ-H--.H--MAYTTQYPWSTRRQD--CQKQ-IVAVRNCC-TTNTDANKRQVIE-DQDQADMGFFRK-TMWL- 736

CPP541714-HNU-CBY-2023 TDSQLKN...LTFRAPVAIGHPQTSDRYAINPLVHQPWSVR..............TED...QDGGSYSVHNYLGGVAYTH 719

EU200668-Bovine_hokovirus-HK3 -------...-------------------------------..............---...----T-------------P 719

JF504698-Bovine_hokovirus_2-HK-B38 -------...-------------------------------..............---...------------------- 719

NC_007018-Human_PARV4 --T-IR-...T---D-----N-A-----SVA----------..............--EW.LANKTD-A-----------R 702

JQ425257-PPV3-US-HK238 ----IR-...TD--V-L---N-V-G---SVG--------I-..............--EG.KSPPNNFA--S--------R 711

JX101462-PPV2-US-523 VST--RASSDFDEMSAI-Y-D-TSNN-HSLM-FTR-ATTIS..............-QNYNR-GEVERN--FQ--DM-FAR 810

AF406966-BPV2 ANRHRDG..DCQIIP-KGREYHVLPV-SGLP-VIVVRQGIFNPLPASGVFGLTSG--QPGPPTEDKAVITPGGTT-LTSN 814

CPP541714-HNU-CBY-2023 RLHEESYDGYQEE......IDGTVSNPSRVVVTDTDLAAPHIGHTFFVPGHQRVSGSG..TETEFNPKLYQEPVFPLFPG 791

EU200668-Bovine_hokovirus-HK3 -------E-----......---------------------------Y-----------..-------------------- 791

JF504698-Bovine_hokovirus_2-HK-B38 -------------......-----T---------------------------------..-------------------- 791

NC_007018-Human_PARV4 -K------KHE-D......R--R-T------QI-G------V---------T--TSG-..-D-VYS-------------- 774

JQ425257-PPV3-US-HK238 -R-----T-HT--......M--S-T-------NEV-M----V----M----T--D---SGSD-VYD-------I-----A 785

JX101462-PPV2-US-523 SSA-D-FYERF--DKDYRNPG-Y-KK-RPL-TAEREGLGERP-DALM--TWGAHLPGSSTGPGTTKTEKVSL-FI-PM-- 890

AF406966-BPV2 T-TVKRKHK-KNQNHDVHTVYEGKQEQK-LYQLVVQRERGVG-PAEPNHVQE-II--TGEKLPGSRYP-QS-ITYGQHT- 894

CPP541714-HNU-CBY-2023 AVWNPNPLSFDCQIWTKIPDTECHFFAQYPLLGGWGMANPPPMIFLKLRAQPGPPSA...GAHTVSKSNLNQYAIFHLHY 868

EU200668-Bovine_hokovirus-HK3 ---------------------------------------------------------...-------------------- 868

JF504698-Bovine_hokovirus_2-HK-B38 ---------------------------------------------------------...-------------------- 868

NC_007018-Human_PARV4 ---------Y---------N----------------VLT------V---S------P...-----PQ------------- 851

JQ425257-PPV3-US-HK238 ---D----TY-------------R-------------DA----V---M-S-----PG...-----PN------------- 862

JX101462-PPV2-US-523 -C-DER--CYEDD--C-K-Y-D-S-MSEKNN--A-ALVD---QV-FRMQP-V---P-D.LDQR-FLPPA-----M-TVS- 969

AF406966-BPV2 --EESESGFYEF---ERN-N-DLGKGGHK-P-AQ-A-EK---T-Y-RMLPM-CA-CKNKYTKSPGM-GVI-S-IT-Q-Q- 974

CPP541714-HNU-CBY-2023 SMEFEVKRRRRSRRHNPEKPAPFPVTESGRMPFTLANDTSDPSKAVYEVPADQWIAQNYSHKL. 931

EU200668-Bovine_hokovirus-HK3 --------------------------------------KT-----------------------. 931

JF504698-Bovine_hokovirus_2-HK-B38 ---------------------------------------T-----------------------. 931

NC_007018-Human_PARV4 --Q-L----K--------------T-D----------SLK--NTP-----S-----R----L-. 914

JQ425257-PPV3-US-HK238 TI-W--------------------T-D------M----DR--NVP-----S---V---F-R--. 925

JX101462-PPV2-US-523 T--WVCEP-KHT----L-P-P-M-Y----DP--L-TRSHASNDYPR-SL-VEAFRPEGRA-RV. 1032

AF406966-BPV2 -IKWAYTP-THT--W--TS--LL-PPLP-STVVYNLDSQKFTTDNQ-TIA-ES-QFK-RLRHNR 1038

**Fig. S2** Amino acid sequence alignment of VP1 with the putative phospholipase A2 motif of the present bovine tetraparvovirus HNU-CBY-2023 and other represent parvoviruses. The Ca^2+^ binding loop is indicated by filled squares and the catalytic residues are indicated by filled circles. ‘‘.’’ indicates a deletion compared to the strain on top. The positions of the amino acids and the GenBank numbers of the sequences are indicated. PPV: porcine parvovirus; BPV: bovine parvovirus.

**Fig.S3** The phylogenetic trees were constructed by using the Neighbor-Joining method based on *p*-distance model, with the nucleotide sequences of ORF2 (VP1) of the present tetraparvovirus ungulate 1 and other represent parvoviruses within genus tetraparvovirus. The percentage of replicate trees in which the associated taxa clustered together in the bootstrap test (1000 replicates) are shown next to the branches (only >70% are shown). The trees are drawn to scale, with branch lengths measured in the number of substitutions per site. The analyses involved 40 sequences of parvoviruses with their GenBank accession numbers indicated. Tetraparvirus ungulate 1 HNU-CBY-2023 characterized in the present study are in bold and marked with “▲”. All positions containing gaps and missing data were eliminated. There were a total of 2720 positions in the final dataset. All positions containing gaps and missing data were eliminated.  Evolutionary analyses were conducted in MEGA7[1].

1. Kumar S, Stecher G, Tamura K: **MEGA7: Molecular Evolutionary Genetics Analysis Version 7.0 for Bigger Datasets**. *Mol Biol Evol* 2016, **33**(7):1870-1874.
